# Crystal structure of hepta­guanidinium nona­hydrogen bis­[α-hexa­molybdoplatinate(IV)] hepta­hydrate

**DOI:** 10.1107/S2056989015002601

**Published:** 2015-02-13

**Authors:** Hea-Chung Joo, Ki-Min Park, Uk Lee

**Affiliations:** a Department of Chemistry, Pukyong National University, 599-1 Daeyeon 3-dong, Nam-gu, Busan 608-737, Republic of Korea; b The Research Institute of Natural Science, Gyeongsan National University, Jinju 660-701, Republic of Korea

**Keywords:** crystal structure, platinum-containing heteropolyoxomolybdate, centrosymmetric hydrogen bond, strong hydrogen bond, Anderson-type heteropolyoxomolybdate

## Abstract

The title compound, (CH_6_N_3_)_7_H_9_[PtMo_6_O_24_]_2_·7H_2_O, containing the well-known Anderson-type heteropolyoxomolybdate, was obtained by recrystallization of its powdered guanidinium salt. The protonated O atoms in the polyanion were confirmed by electron-density maps, inter­polyanion hydrogen bonds and bond-valance sums (BVS). The {[H_4.5_PtMo_6_O_24_]_2_}^7−^ polyanion is the same as that already characterized in K_7_[H_4.5_PtMo_6_O_24_]_2_·11H_2_O [space group *P*-1; Lee & Joo (2010 ▸). *Acta Cryst.* E**66**, i8–i9]. The heteropolyanions form inversion-generated dimers, {[H_4.5_PtMo_6_O_24_]_2_}^7−^, held together by each of the four μ_3_-O—H⋯μ_1_-O, two μ_2_-O—H⋯μ_2_-O hydrogen bonds and one centrosymmetric μ_3_-O—H—μ_3_-O hydrogen bond. The H atom of the centrosymmetric hydrogen bond is located on an inversion centre. One guanidinium ion and one water mol­ecule are equally disordered about a twofold rotation axis.

## Abstract 2

A compound, (CH_6_N_3_)_7_H_9_[PtMo_6_O_24_]_2_·7H_2_O, containing the well-known Anderson-structure heteropolyoxomolybdate, was obtained by recrystallization of its powdered guanidinium salt. The protonated O atoms in the polyanion were confirmed by electron-density maps, inter­polyanion hydrogen bonds and bond-valance sums (BVS).

## Chemical context   

The *α* (planar structure)-*β* (bent structure)-*α* geometrical isomerization, according to stepwise protonation in the [PtMo_6_O_24_]^8−^ polyoxometalate (POM) species, *viz*. ([H_3.5_ *α*-PtMo_6_O_24_]^4.5−^ (Lee & Sasaki, 1994 ▸), [H_4_ *β*-PtMo_6_O_24_]^4−^ (Lee & Sasaki, 1994 ▸; Joo *et al.*, 1994 ▸) and [H_4.5_ *α*-PtMo_6_O_24_]^3.5−^ (Lee & Sasaki, 1994 ▸; Lee *et al.*, 2010 ▸) is an unprecedented phenomenon in the Anderson-type heteropolyanion (Anderson, 1937 ▸), as well as in the chemistry of polyoxo­metalates.
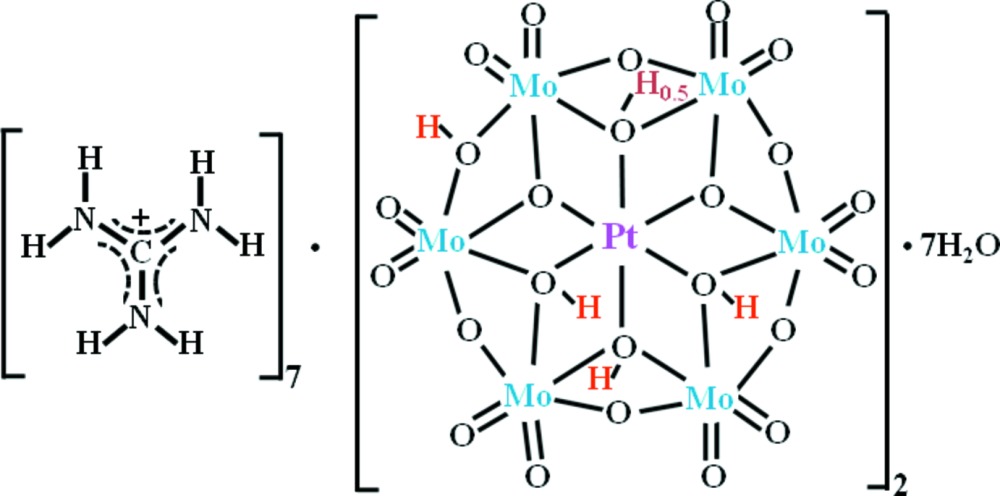


As a result of the insolubility of the guanidinium salt, replaceable counter-cations in POMs can be exchanged by guanidinium ions. It is thus possible to obtain stable POMs by precipitation from aqueous solution with guanidinium salts. The guanidinium salts of platinum-containing POM species, *viz*. (CH_6_N_3_)_8_[PtW_6_O_24_] (Lee *et al.*, 2003 ▸ ▸), (CH_6_N_3_)_5_[H_2_PtV_9_O_28_] (Joo *et al.*, 2011 ▸) and (CH_6_N_3_)_8_[α-SiPt_2_W_10_O_40_]·6H_2_O (Lee *et al.*, 2003 ▸ ▸) have been reported by our group. The positions of the protonated O atoms in the {[H_4.5_α-PtMo_6_O_24_]_2_}^7−^ polyanion were reconfirmed in the present study.

Sometimes a short hydrogen bond, O⋯O distance < 2.60 Å, in which the H atom lies on a crystallographic center of symmetry, occurs in this class of structure. The H atom of the central hydrogen bond, O6*C*—H6—O6*C* ^i^ in the title compound lies on a crystallographic center of symmetry (space group *C*2/*c*: 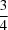, 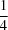, 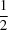).

## Structural commentary   

**Figure 1:**
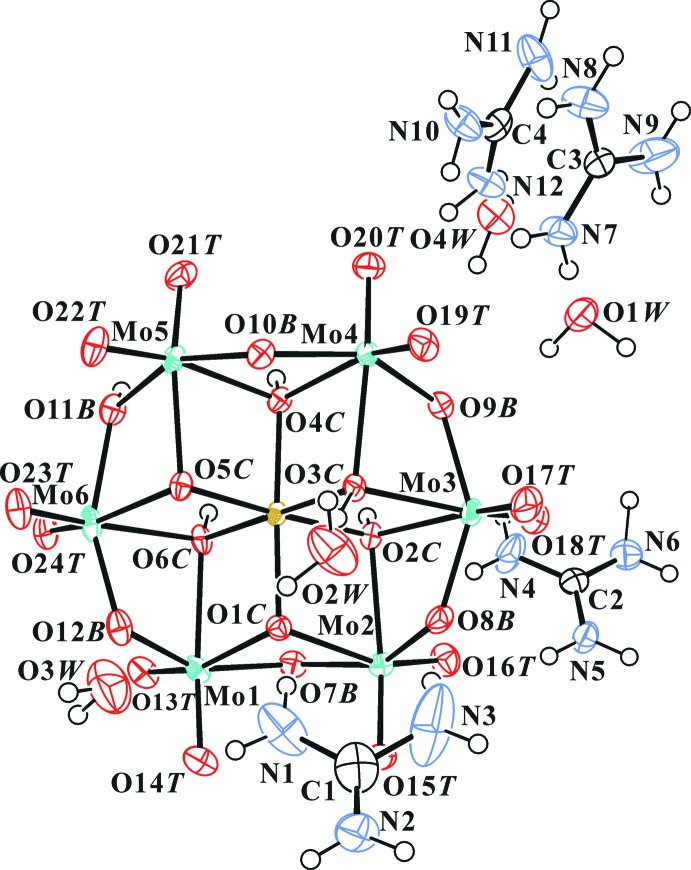
The mol­ecular structure of the title compound, showing the atom-numbering scheme. Displacement ellipsoids are drawn at the 50% probability level. H atoms are presented as small spheres of arbitrary radius. Disordered parts have been omitted for clarity.

**Figure 2:**
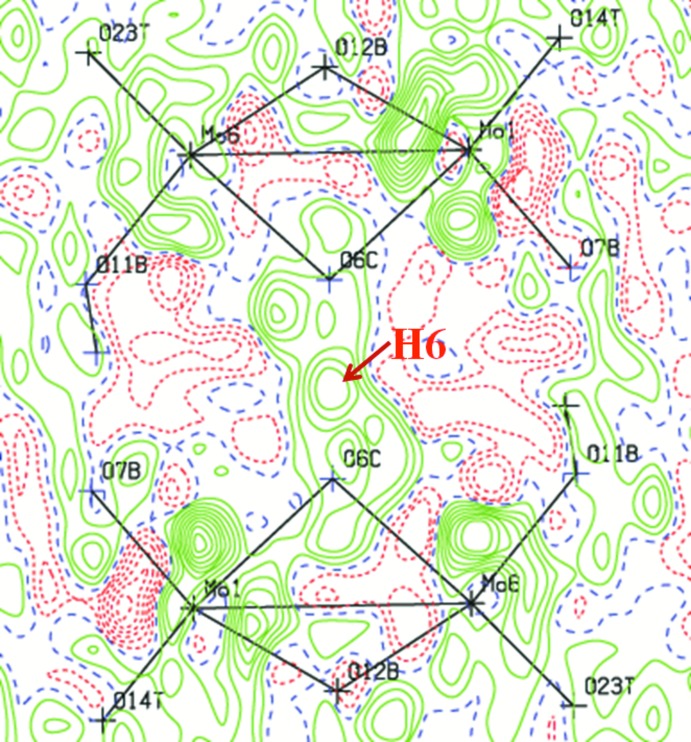
Difference-Fourier map around atom H6 (calculated with atom H6 absent from the model).

**Figure 3:**
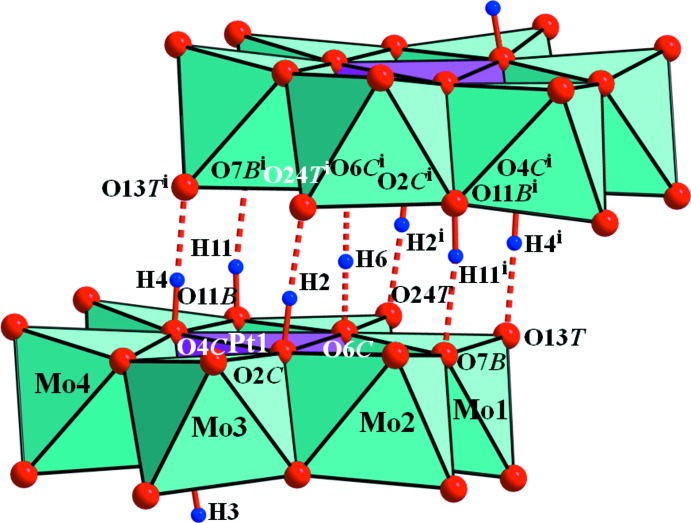
Polyhedral view of the heteropolyanion in the title compound with O—H⋯O contacts of the inter­anion hydrogen bonds shown as red dashed lines. [Symmetry code: (i) −*x* + 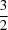, −*y* + 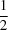, −*z* + 1.]

The structure of the title compound POM anion has been discussed in detail (Lee *et al.*, 2010 ▸). Fig. 1 ▸ shows the structure of the title compound, and selected geometrical parameters are given in Table 1 ▸. The complete polyanion has *C*_1_ (1) symmetry. The O atoms of the heteropolyanion have been designated as O*T* (terminal Mo=O atom), O*B* (bridging μ_2_-O atom), and O*C* (centered μ_3_-O atom). The protonated O atoms in the polyanion were confirmed in electron density maps, inter­polyanion hydrogen bonds (Table 2 ▸) and by bond-valence sums (BVS; Brown & Altermatt, 1985 ▸; Brese & O’Keeffe, 1991 ▸). Fig. 2 ▸ shows a symmetric electron-density map around the position of atom H6. The H atom of the centrosymmetric hydrogen bond in the compound lies on a crystallographic centre of symmetry (space group *C*2/*c*: 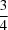, 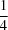, 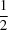). The O6*C*—H6 and O6*C*⋯O6*C* ^i^ distances are 1.27 and 2.532 (6) Å, and the O6*C*—H6—O6*C* ^i^ angle is 180° (Table 2 ▸ and Fig. 3 ▸). Atom H3 does not contribute to dimer formation because it is located on the other side of the polyanion.

Confirmation of the protonated O atoms was strongly supported by the BVS analysis. The calculated BVS for atoms O2*C*, O3*C*, O4*C*, O6*C* and O11*B* are 1.40, 1.36, 1.38, 1.41 and 1.30 valence units (v.u.), respectively, if the valence of the O—H bond is not included. Since the BVS value around the μ_2_-O atom should be 2.0 v.u., the missing valences of O2*C*, O3*C*, O4*C*, O6*C* and O11*B* are 0.60, 0.64, 0.62, 0.59 and 0.70 v.u., respectively, which corresponds to the valence of the O—H bonds. The BVS value range for the unprotonated O*C* and O*B* atoms is 1.68–1.90 v.u. As a result, the protonated O atoms were O2*C*, O3*C*, O4*C*, O11*B* and O6*C*. The protonated features of both the {[H_4.5_PtMo_6_O_24_]_2_}^7−^ polyanion in the title compound and in K_7_[H_4.5_PtMo_6_O_24_]_2_.11H_2_O (space group *P* 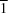) are exactly the same. The bond lengths and bond angles involving protonated and unprotonated O atoms in the {[H_4.5_PtMo_6_O_24_]_2_}^7−^ polyanion are compared in Table 1 ▸. The Pt—O*C* bond lengths were not affected by protonation of the O*C* atoms.

The C4 guanidinium ion and O4*W* water mol­ecule are equally disordered about a twofold rotation axis.

## Supra­molecular features   

The heteropolyanions form inversion-generated dimers, {[H_4.5_PtMo_6_O_24_]_2_}^7−^ held together by each of the four μ_3_-O—H⋯μ_1_-O (terminal O atom), two μ_2_-O—H⋯μ_2_-O and one centrosymmetric μ_3_-O—H—μ_3_-O hydrogen bonds (Table 2 ▸). Furthermore, the polyanions are linked in three dimensions *via* N—H⋯O hydrogen bonds. All water mol­ecules form hydrogen bonds with O atoms of the polyanions except for the O2*W* water mol­ecule (Table 2 ▸). Hydrogen-bonding interactions involving the disordered molecules have been omitted.

## Database survey   

A number of Anderson-structure platinum(IV)-containing heteropolyoxomolybdates have been reported: [H_4.5_PtMo_6_O_24_]^3.5−^ and [H_4_PtMo_6_O_24_]^4−^, [H_3.5_PtMo_6_O_24_]^4.5−^ (Lee & Sasaki, 1994 ▸); [H_4_β-PtM0_6_0_24_]^4−^ (Joo *et al.*, 1994 ▸); [H_2_PtMo_6_O_24_]^6−^ (Lee & Joo, 2000 ▸, 2004 ▸); [H_4.5_PtMo_6_O_24_]^3.5−^ (Lee *et al.*, 2010 ▸); [H_6_PtMo_6_O_24_]^2−^ (Lee & Joo, 2010 ▸); [H_23_(PtMo_6_O_24_)_4_]^9−^, [H_16_(PtMo_6_O_24_)_3_]^8−^ and [H_14_(PtMo_6_O_24_)_3_]^14−^ (Day *et al.*, 2009 ▸).

## Synthesis and crystallization   

A pale-yellow powder of the title compound was obtained by addition of a small excess of the stoichiometric qu­antity of guanidinium chloride, CH_6_N_3_Cl, to a solution of the sodium salt of hexa­molybdoplatinate hydrate. Single crystals were obtained by recrystallization from a hot aqueous solution of the crude sample in an insulating chamber.

## Refinement   

Crystal data, data collection and structure refinement details are summarized in Table 3 ▸. All the H atoms in the polyanion and all water H atoms were positioned using difference Fourier maps. All H atoms of the polyanion were refined with a distance restraint of O—H = 0.95 (2) Å using the DFIX command (Sheldrick, 2008 ▸). All H atoms of the guanidinium ions were positioned geometrically and refined using a riding model, with *U*_iso_(H) = 1.5*U*_eq_(N). The C4 guanidinium ion and O4*W* water mol­ecule are equally disordered about a twofold rotation axis. Refinement of the site occupation factors (s.o.f) converged at values close to half occupancy. In the final refinement, the s.o.f.s were constrained to 0.5 and reasonable displacement parameters were obtained. The C—N and N—H bond lengths were restrained to 1.30 (2) and 0.90 (2) Å, respectively, and the H*A*—N—H*B* angles were restrained by restraining the H*A*⋯H*B* distance to 1.55 (2) Å in the disordered C4 guanidinium ion using the DFIX command. The H atoms of all water mol­ecules (O*W*) were refined with a distance restraint of O—H = 0.95 (2) Å using the DFIX, and were included in the refinement with *U*_iso_(H) = 1.5*U*_eq_(O). The highest peak in the difference map is 0.98 Å from atom Pt1 and the largest hole is 0.36 Å from N3.

## Supplementary Material

Crystal structure: contains datablock(s) New_Global_Publ_Block, I. DOI: 10.1107/S2056989015002601/pk2544sup1.cif

Structure factors: contains datablock(s) I. DOI: 10.1107/S2056989015002601/pk2544Isup2.hkl

CCDC reference: 1048266

Additional supporting information:  crystallographic information; 3D view; checkCIF report

## Figures and Tables

**Table 1:** Selected geometric parameters (, )

Pt1O1*C*	1.995(3)	Mo5O5*C*	2.178(3)
Pt1O2*C*	2.015(3)	Mo6O5*C*	2.123(3)
Pt1O3*C*	2.027(3)	Mo6O6*C*	2.277(3)
Pt1O4*C*	2.011(3)	Mo1O7*B*	1.965(3)
Pt1O5*C*	1.997(3)	Mo1O12*B*	1.959(3)
Pt1O6*C*	2.005(3)	Mo2O7*B*	1.978(3)
Mo1O1*C*	2.150(3)	Mo2O8*B*	1.945(3)
Mo1O6*C*	2.317(3)	Mo3O8*B*	1.934(3)
Mo2O1*C*	2.248(3)	Mo3O9*B*	1.952(3)
Mo2O2*C*	2.286(3)	Mo4O9*B*	1.941(3)
Mo3O2*C*	2.307(3)	Mo4O10*B*	1.959(3)
Mo3O3*C*	2.318(3)	Mo5O10*B*	1.895(3)
Mo4O3*C*	2.287(3)	Mo5O11*B*	2.058(3)
Mo4O4*C*	2.327(3)	Mo6O11*B*	2.075(4)
Mo5O4*C*	2.289(3)	Mo6O12*B*	1.894(4)
			
Mo1O1*C*Mo2	95.79(12)	Mo1O7*B*Mo2	111.71(15)
Mo2O2*C*Mo3	93.64(11)	Mo3O8*B*Mo2	119.36(16)
Mo4O3*C*Mo3	93.75(12)	Mo4O9*B*Mo3	119.39(17)
Mo5O4*C*Mo4	92.64(11)	Mo5O10*B*Mo4	120.02(16)
Mo6O5*C*Mo5	102.87(13)	Mo5O11*B*Mo6	108.97(15)
Mo6O6*C*Mo1	91.14(12)	Mo6O12*B*Mo1	116.75(17)

**Table 2:** Hydrogen-bond geometry (, )

*D*H*A*	*D*H	H*A*	*D* *A*	*D*H*A*
O2*C*H2O24*T* ^i^	0.96(2)	1.61(2)	2.578(5)	179(6)
O3*C*H3O2*W*	0.96(2)	1.69(3)	2.622(6)	164(7)
O4*C*H4O13*T* ^i^	0.95(2)	1.63(2)	2.568(5)	173(9)
O6*C*H6O6*C* ^i^	1.27	1.27	2.532(6)	180
O11*B*H11O7*B* ^i^	0.95(2)	1.74(2)	2.679(5)	173(10)
N1H1*B*O1*C*	0.88	2.05	2.864(6)	154
N1H1*A*O3*W*	0.88	2.33	2.973(9)	130
N2H2*A*O18*T* ^ii^	0.88	2.08	2.940(7)	165
N2H2*B*O19*T* ^iii^	0.88	2.22	3.043(6)	155
N3H3*B*O8*B*	0.88	2.04	2.874(7)	157
N3H3*A*O2*W* ^iii^	0.88	2.25	2.979(9)	140
N4H4*B*O14*T* ^iv^	0.88	2.09	2.944(6)	164
N4H4*A*O24*T* ^i^	0.88	2.48	3.006(6)	119
N5H5*A*O16*T*	0.88	2.06	2.890(6)	157
N5H5*B*O21*T* ^v^	0.88	2.18	2.973(5)	149
N6H6*A*O15*T* ^iv^	0.88	2.19	2.894(6)	136
N6H6*B*O21*T* ^v^	0.88	2.59	3.281(6)	136
N7H7*B*O19*T*	0.88	2.40	2.936(5)	119
N7H7*A*O1*W*	0.88	2.11	2.927(6)	154
N8H8*B*O13*T* ^vi^	0.88	2.39	3.006(6)	128
N8H8*A*O23*T* ^vii^	0.88	2.04	2.918(6)	178
N9H9*A*O22*T* ^vii^	0.88	2.21	2.938(7)	140
O1*W*H1*AW*O9*B*	0.94(2)	2.20(5)	2.916(5)	132(5)
O1*W*H1*BW*O17*T* ^viii^	0.95(2)	1.85(3)	2.783(5)	166(6)
O2*W*H2*BW*O4*W* ^ii^	0.95(2)	2.24(7)	2.902(12)	126(6)
O3*W*H3*BW*O9*B* ^ii^	0.94(2)	2.35(8)	3.029(7)	128(8)

Symmetry codes: (i) 
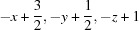
; (ii) 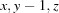; (iii) 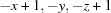; (iv) 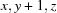; (v) 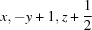; (vi) 
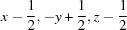
; (vii) 
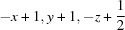
; (viii) 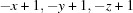.

**Table 3:** Experimental details

Crystal data	
Chemical formula	(CH_6_N_3_)_7_H_9_[PtMo_6_O_24_]_2_7H_2_O
*M*_r_	2865.26
Crystal system, space group	Monoclinic, *C*2/*c*
Temperature (K)	173
*a*, *b*, *c* ()	31.413(10), 10.073(3), 23.677(7)
()	119.451(14)
*V* (^3^)	6524(3)
*Z*	4
Radiation type	Mo *K*
(mm^1^)	6.62
Crystal size (mm)	0.30 0.12 0.05

Data collection	
Diffractometer	Bruker *SMART* APEXII CCD
Absorption correction	Multi-scan (*SADABS*; Sheldrick, 2008 ▸)
*T*_min_, *T*_max_	0.241, 0.729
No. of measured, independent and observed [*I* > 2(*I*)] reflections	56606, 7107, 6050
*R*_int_	0.033
(sin /)_max_ (^1^)	0.639

Refinement	
*R*[*F* ^2^ > 2(*F* ^2^)], *wR*(*F* ^2^), *S*	0.028, 0.073, 1.03
No. of reflections	7107
No. of parameters	505
No. of restraints	22
H-atom treatment	H atoms treated by a mixture of independent and constrained refinement
_max_, _min_ (e ^3^)	2.50, 1.30

Computer programs: *APEX2* and *SAINT* (Bruker, 2009 ▸), *SHELXS97* and *SHELXL97* (Sheldrick, 2008 ▸), *ORTEP-3 for Windows* (Farrugia, 2012 ▸), *PLATON* (Spek, 2009 ▸) and *DIAMOND* (Brandenburg, 1998 ▸).

## supplementary crystallographic information

### Crystal data

**Table:**

(CH_6_N_3_)_7_H_9_[PtMo_6_O_24_]_2_·7H_2_O	*F*(000) = 5416
*M**_r_* = 2865.26	*D*_x_ = 2.917 Mg m^−^^3^
Monoclinic, *C*2/*c*	Mo *K*α radiation, λ = 0.71073 Å
Hall symbol: -C 2yc	Cell parameters from 9569 reflections
*a* = 31.413 (10) Å	θ = 2.2–28.2°
*b* = 10.073 (3) Å	µ = 6.62 mm^−^^1^
*c* = 23.677 (7) Å	*T* = 173 K
β = 119.451 (14)°	Block, yellow
*V* = 6524 (3) Å^3^	0.30 × 0.12 × 0.05 mm
*Z* = 4	

### Data collection

**Table:**

Bruker SMART APEXII CCD diffractometer	7107 independent reflections
Radiation source: Rotating Anode	6050 reflections with *I* > 2σ(*I*)
Graphite multilayer monochromator	*R*_int_ = 0.033
Detector resolution: 10.0 pixels mm^-1^	θ_max_ = 27.0°, θ_min_ = 1.5°
φ and ω scans	*h* = −40→36
Absorption correction: multi-scan (*SADABS*; Sheldrick, 2008)	*k* = −12→12
*T*_min_ = 0.241, *T*_max_ = 0.729	*l* = −30→30
56606 measured reflections	

### Refinement

**Table:**

Refinement on *F*^2^	Primary atom site location: structure-invariant direct methods
Least-squares matrix: full	Secondary atom site location: difference Fourier map
*R*[*F*^2^ > 2σ(*F*^2^)] = 0.028	Hydrogen site location: difference Fourier map
*wR*(*F*^2^) = 0.073	H atoms treated by a mixture of independent and constrained refinement
*S* = 1.03	*w* = 1/[σ^2^(*F*_o_^2^) + (0.0337*P*)^2^ + 47.9084*P*] where *P* = (*F*_o_^2^ + 2*F*_c_^2^)/3
7107 reflections	(Δ/σ)_max_ = 0.002
505 parameters	Δρ_max_ = 2.50 e Å^−^^3^
22 restraints	Δρ_min_ = −1.30 e Å^−^^3^

### Special details

**Table:**

Geometry. All esds (except the esd in the dihedral angle between two l.s. planes) are estimated using the full covariance matrix. The cell esds are taken into account individually in the estimation of esds in distances, angles and torsion angles; correlations between esds in cell parameters are only used when they are defined by crystal symmetry. An approximate (isotropic) treatment of cell esds is used for estimating esds involving l.s. planes.
Refinement. Refinement of F^2^ against ALL reflections. The weighted R-factor wR and goodness of fit S are based on F^2^, conventional R-factors R are based on F, with F set to zero for negative F^2^. The threshold expression of F^2^ > 2sigma(F^2^) is used only for calculating R-factors(gt) etc. and is not relevant to the choice of reflections for refinement. R-factors based on F^2^ are statistically about twice as large as those based on F, and R- factors based on ALL data will be even larger.

### Fractional atomic coordinates and isotropic or equivalent isotropic displacement parameters (Å^2^)

**Table:**

	*x*	*y*	*z*	*U*_iso_*/*U*_eq_	Occ. (<1)
Pt1	0.652431 (6)	0.176198 (17)	0.467608 (8)	0.01525 (6)	
Mo1	0.743430 (14)	−0.00319 (4)	0.57409 (2)	0.02093 (10)	
Mo2	0.682566 (15)	0.17866 (4)	0.624195 (19)	0.02035 (10)	
Mo3	0.583929 (15)	0.34871 (4)	0.51759 (2)	0.02171 (10)	
Mo4	0.552026 (14)	0.34729 (4)	0.359683 (19)	0.01849 (9)	
Mo5	0.618484 (14)	0.17217 (4)	0.311229 (19)	0.01901 (10)	
Mo6	0.712270 (14)	−0.01255 (4)	0.42001 (2)	0.02259 (10)	
O1C	0.66821 (11)	0.0483 (3)	0.53959 (14)	0.0194 (7)	
O2C	0.66214 (11)	0.3107 (3)	0.53597 (15)	0.0187 (7)	
H2	0.6862 (17)	0.378 (5)	0.544 (3)	0.050 (18)*	
O3C	0.58105 (11)	0.1962 (3)	0.44249 (15)	0.0178 (7)	
H3	0.568 (2)	0.109 (3)	0.439 (3)	0.07 (2)*	
O4C	0.63463 (11)	0.3099 (3)	0.39634 (15)	0.0184 (7)	
H4	0.656 (3)	0.379 (6)	0.398 (4)	0.10 (3)*	
O5C	0.64084 (11)	0.0488 (3)	0.39683 (14)	0.0191 (7)	
O6C	0.72274 (11)	0.1511 (3)	0.49205 (15)	0.0190 (7)	
H6	0.7500	0.2500	0.5000	0.06 (3)*	
O7B	0.74507 (12)	0.1539 (3)	0.62431 (16)	0.0229 (7)	
O8B	0.61194 (12)	0.2010 (3)	0.57683 (16)	0.0243 (7)	
O9B	0.57705 (11)	0.4420 (3)	0.44128 (15)	0.0226 (7)	
O10B	0.55913 (11)	0.1971 (3)	0.31241 (15)	0.0212 (7)	
O11B	0.69300 (12)	0.1458 (3)	0.35603 (16)	0.0235 (7)	
H11	0.713 (3)	0.222 (7)	0.362 (5)	0.15 (4)*	
O12B	0.71759 (12)	−0.1040 (3)	0.49309 (17)	0.0262 (8)	
O13T	0.80360 (12)	0.0093 (3)	0.59080 (17)	0.0285 (8)	
O14T	0.74286 (12)	−0.1258 (3)	0.62346 (17)	0.0305 (8)	
O15T	0.68520 (13)	0.0508 (4)	0.67282 (16)	0.0299 (8)	
O16T	0.69977 (13)	0.3193 (3)	0.67096 (17)	0.0312 (8)	
O17T	0.52417 (13)	0.3226 (4)	0.49587 (19)	0.0371 (9)	
O18T	0.60285 (14)	0.4814 (4)	0.56903 (17)	0.0370 (9)	
O19T	0.49288 (12)	0.3228 (3)	0.34110 (17)	0.0275 (8)	
O20T	0.55233 (12)	0.4783 (3)	0.31420 (16)	0.0283 (8)	
O21T	0.62270 (13)	0.3112 (4)	0.27214 (17)	0.0292 (8)	
O22T	0.60336 (13)	0.0433 (4)	0.25787 (17)	0.0315 (8)	
O23T	0.69102 (12)	−0.1349 (3)	0.36251 (18)	0.0304 (8)	
O24T	0.77302 (12)	0.0093 (3)	0.44119 (17)	0.0290 (8)	
C1	0.5886 (2)	−0.1669 (7)	0.5871 (3)	0.0536 (18)	
N1	0.6167 (2)	−0.1725 (5)	0.5534 (3)	0.0611 (18)	
H1A	0.6273	−0.2493	0.5476	0.073*	
H1B	0.6230	−0.0991	0.5388	0.073*	
N2	0.57817 (17)	−0.2673 (5)	0.6092 (2)	0.0446 (13)	
H2A	0.5879	−0.3464	0.6046	0.053*	
H2B	0.5611	−0.2586	0.6293	0.053*	
N3	0.5678 (3)	−0.0394 (7)	0.5888 (4)	0.106 (3)	
H3A	0.5480	−0.0333	0.6052	0.127*	
H3B	0.5748	0.0317	0.5735	0.127*	
C2	0.69992 (17)	0.6537 (5)	0.7035 (2)	0.0252 (11)	
N4	0.7180 (2)	0.6254 (5)	0.6658 (3)	0.0482 (14)	
H4A	0.7210	0.5420	0.6571	0.058*	
H4B	0.7273	0.6896	0.6490	0.058*	
N5	0.68617 (16)	0.5573 (4)	0.7284 (2)	0.0310 (10)	
H5A	0.6892	0.4741	0.7196	0.037*	
H5B	0.6739	0.5760	0.7538	0.037*	
N6	0.69567 (17)	0.7785 (5)	0.7166 (2)	0.0355 (11)	
H6A	0.7050	0.8426	0.6999	0.043*	
H6B	0.6835	0.7976	0.7420	0.043*	
C3	0.4036 (2)	0.6616 (5)	0.2726 (3)	0.0290 (11)	
N7	0.43204 (15)	0.5625 (4)	0.3073 (2)	0.0324 (10)	
H7A	0.4546	0.5757	0.3479	0.039*	
H7B	0.4284	0.4834	0.2898	0.039*	
N8	0.36976 (18)	0.6419 (5)	0.2123 (2)	0.0444 (13)	
H8A	0.3507	0.7078	0.1894	0.053*	
H8B	0.3661	0.5628	0.1947	0.053*	
N9	0.4097 (3)	0.7778 (5)	0.2998 (3)	0.075 (2)	
H9A	0.3910	0.8448	0.2775	0.090*	
H9B	0.4326	0.7894	0.3403	0.090*	
C4	0.5228 (3)	0.8412 (9)	0.2828 (4)	0.028 (2)	0.50
N10	0.5000	0.7313 (6)	0.2500	0.0368 (16)	
H10A	0.4775 (14)	0.686 (4)	0.2159 (13)	0.044*	
N11	0.5000	0.9567 (6)	0.2500	0.0471 (19)	
H11A	0.5223 (16)	1.002 (4)	0.2844 (14)	0.057*	
N12	0.5653 (4)	0.8331 (14)	0.3368 (6)	0.039 (3)	0.50
H12A	0.579 (3)	0.753 (5)	0.343 (6)	0.046*	0.50
H12B	0.584 (3)	0.905 (6)	0.355 (6)	0.046*	0.50
O1W	0.51593 (15)	0.6741 (4)	0.42228 (19)	0.0400 (10)	
H1AW	0.5475 (12)	0.638 (6)	0.446 (3)	0.060*	
H1BW	0.507 (2)	0.678 (6)	0.456 (2)	0.060*	
O2W	0.52988 (18)	−0.0205 (5)	0.4254 (3)	0.0609 (13)	
H2AW	0.508 (3)	−0.006 (8)	0.3811 (14)	0.091*	
H2BW	0.553 (2)	−0.091 (6)	0.435 (4)	0.091*	
O3W	0.6421 (2)	−0.3187 (6)	0.4650 (3)	0.0764 (17)	
H3AW	0.6747 (13)	−0.346 (9)	0.487 (4)	0.115*	
H3BW	0.632 (4)	−0.398 (6)	0.441 (4)	0.115*	
O4W	0.5802 (4)	0.8321 (11)	0.3704 (5)	0.036 (2)	0.50
H4AW	0.599 (4)	0.911 (8)	0.381 (6)	0.055*	0.50
H4BW	0.602 (4)	0.770 (10)	0.402 (5)	0.055*	0.50

### Atomic displacement parameters (Å^2^)

**Table:**

	*U*^11^	*U*^22^	*U*^33^	*U*^12^	*U*^13^	*U*^23^
Pt1	0.01074 (9)	0.01946 (10)	0.01597 (9)	−0.00051 (6)	0.00690 (7)	−0.00191 (7)
Mo1	0.01368 (19)	0.0186 (2)	0.0308 (2)	0.00080 (15)	0.01116 (17)	0.00340 (17)
Mo2	0.0199 (2)	0.0212 (2)	0.0193 (2)	−0.00066 (16)	0.00908 (17)	0.00091 (16)
Mo3	0.0190 (2)	0.0282 (2)	0.0213 (2)	0.00438 (17)	0.01255 (17)	−0.00020 (17)
Mo4	0.01413 (19)	0.0230 (2)	0.01809 (19)	0.00203 (15)	0.00773 (16)	0.00188 (16)
Mo5	0.0170 (2)	0.0242 (2)	0.01814 (19)	−0.00386 (16)	0.01039 (17)	−0.00427 (16)
Mo6	0.01442 (19)	0.0212 (2)	0.0358 (2)	−0.00414 (16)	0.01523 (18)	−0.01035 (18)
O1C	0.0188 (16)	0.0194 (17)	0.0189 (15)	0.0004 (13)	0.0083 (13)	−0.0001 (13)
O2C	0.0175 (16)	0.0200 (17)	0.0203 (16)	−0.0025 (13)	0.0107 (14)	−0.0044 (13)
O3C	0.0110 (15)	0.0224 (17)	0.0212 (16)	0.0003 (13)	0.0089 (13)	−0.0007 (13)
O4C	0.0157 (16)	0.0218 (17)	0.0194 (16)	−0.0030 (13)	0.0099 (13)	−0.0016 (13)
O5C	0.0160 (15)	0.0227 (17)	0.0195 (15)	−0.0018 (13)	0.0093 (13)	−0.0053 (13)
O6C	0.0086 (14)	0.0223 (17)	0.0225 (16)	0.0019 (12)	0.0049 (13)	−0.0056 (13)
O7B	0.0182 (16)	0.0249 (18)	0.0228 (17)	−0.0009 (14)	0.0077 (14)	0.0004 (14)
O8B	0.0213 (17)	0.0329 (19)	0.0235 (17)	−0.0006 (15)	0.0149 (15)	0.0021 (15)
O9B	0.0224 (17)	0.0214 (17)	0.0241 (16)	0.0033 (14)	0.0116 (14)	0.0006 (14)
O10B	0.0144 (15)	0.0283 (18)	0.0201 (16)	−0.0035 (13)	0.0079 (13)	−0.0024 (14)
O11B	0.0180 (16)	0.0287 (18)	0.0271 (17)	−0.0031 (14)	0.0135 (15)	−0.0036 (15)
O12B	0.0200 (17)	0.0226 (18)	0.041 (2)	−0.0029 (14)	0.0185 (16)	−0.0060 (15)
O13T	0.0204 (17)	0.0244 (18)	0.038 (2)	−0.0017 (14)	0.0124 (16)	0.0006 (15)
O14T	0.0245 (18)	0.0262 (19)	0.038 (2)	0.0018 (15)	0.0131 (16)	0.0075 (16)
O15T	0.0318 (19)	0.032 (2)	0.0264 (18)	0.0031 (16)	0.0144 (16)	0.0078 (16)
O16T	0.030 (2)	0.029 (2)	0.0307 (19)	0.0001 (15)	0.0115 (17)	−0.0062 (15)
O17T	0.0255 (19)	0.057 (3)	0.037 (2)	0.0066 (17)	0.0210 (18)	0.0066 (18)
O18T	0.048 (2)	0.036 (2)	0.0266 (19)	0.0097 (18)	0.0184 (18)	−0.0030 (17)
O19T	0.0159 (16)	0.033 (2)	0.0313 (19)	0.0022 (14)	0.0099 (15)	0.0011 (15)
O20T	0.0261 (18)	0.031 (2)	0.0254 (17)	0.0023 (15)	0.0112 (15)	0.0051 (15)
O21T	0.0292 (19)	0.038 (2)	0.0257 (18)	−0.0018 (16)	0.0173 (16)	0.0027 (15)
O22T	0.0311 (19)	0.038 (2)	0.0292 (18)	−0.0069 (17)	0.0176 (16)	−0.0152 (16)
O23T	0.0255 (18)	0.0244 (18)	0.044 (2)	−0.0039 (15)	0.0197 (17)	−0.0126 (17)
O24T	0.0257 (18)	0.0280 (19)	0.040 (2)	0.0018 (15)	0.0208 (17)	−0.0045 (16)
C1	0.035 (3)	0.063 (5)	0.052 (4)	−0.005 (3)	0.013 (3)	−0.013 (3)
N1	0.035 (3)	0.035 (3)	0.100 (5)	−0.006 (2)	0.024 (3)	0.000 (3)
N2	0.035 (3)	0.049 (3)	0.057 (3)	−0.003 (2)	0.029 (3)	0.012 (3)
N3	0.134 (7)	0.078 (5)	0.185 (9)	−0.068 (5)	0.140 (7)	−0.066 (5)
C2	0.019 (2)	0.030 (3)	0.021 (2)	−0.002 (2)	0.006 (2)	0.000 (2)
N4	0.075 (4)	0.040 (3)	0.063 (3)	−0.011 (3)	0.059 (3)	−0.005 (3)
N5	0.038 (3)	0.029 (2)	0.035 (2)	−0.002 (2)	0.026 (2)	−0.003 (2)
N6	0.038 (3)	0.032 (3)	0.038 (3)	0.000 (2)	0.021 (2)	0.003 (2)
C3	0.034 (3)	0.030 (3)	0.028 (3)	0.004 (2)	0.019 (2)	0.005 (2)
N7	0.031 (2)	0.032 (2)	0.026 (2)	0.003 (2)	0.0069 (19)	0.0025 (19)
N8	0.038 (3)	0.044 (3)	0.036 (3)	0.005 (2)	0.006 (2)	0.010 (2)
N9	0.154 (7)	0.029 (3)	0.045 (3)	0.016 (4)	0.050 (4)	0.004 (3)
C4	0.022 (5)	0.039 (6)	0.031 (6)	−0.007 (4)	0.019 (5)	−0.002 (5)
N10	0.033 (4)	0.022 (3)	0.045 (4)	0.000	0.012 (3)	0.000
N11	0.042 (4)	0.019 (3)	0.083 (6)	0.000	0.033 (4)	0.000
N12	0.035 (8)	0.030 (6)	0.043 (7)	0.007 (5)	0.013 (6)	0.011 (7)
O1W	0.039 (2)	0.055 (3)	0.031 (2)	0.0063 (19)	0.0207 (19)	0.0038 (18)
O2W	0.051 (3)	0.037 (3)	0.091 (4)	0.000 (2)	0.032 (3)	0.006 (3)
O3W	0.069 (4)	0.072 (4)	0.097 (5)	0.027 (3)	0.048 (4)	0.009 (3)
O4W	0.025 (6)	0.034 (6)	0.050 (7)	−0.010 (4)	0.018 (5)	0.001 (6)

### Geometric parameters (Å, º)

**Table:**

Pt1—O1C	1.995 (3)	O6C—O6C^i^	2.532 (6)
Pt1—O2C	2.015 (3)	O6C—H6	1.266 (3)
Pt1—O3C	2.027 (3)	O11B—H11	0.95 (2)
Pt1—O4C	2.011 (3)	C1—N2	1.254 (8)
Pt1—O5C	1.997 (3)	C1—N1	1.451 (10)
Pt1—O6C	2.005 (3)	C1—N3	1.451 (10)
Mo1—O1C	2.150 (3)	N1—H1A	0.8800
Mo1—O6C	2.317 (3)	N1—H1B	0.8800
Mo2—O1C	2.248 (3)	N2—H2A	0.8800
Mo2—O2C	2.286 (3)	N2—H2B	0.8800
Mo3—O2C	2.307 (3)	N3—H3A	0.8800
Mo3—O3C	2.318 (3)	N3—H3B	0.8800
Mo4—O3C	2.287 (3)	C2—N4	1.304 (7)
Mo4—O4C	2.327 (3)	C2—N5	1.314 (6)
Mo5—O4C	2.289 (3)	C2—N6	1.317 (7)
Mo5—O5C	2.178 (3)	N4—H4A	0.8800
Mo6—O5C	2.123 (3)	N4—H4B	0.8800
Mo6—O6C	2.277 (3)	N5—H5A	0.8800
Mo1—O7B	1.965 (3)	N5—H5B	0.8800
Mo1—O12B	1.959 (3)	N6—H6A	0.8800
Mo2—O7B	1.978 (3)	N6—H6B	0.8800
Mo2—O8B	1.945 (3)	C3—N9	1.303 (7)
Mo3—O8B	1.934 (3)	C3—N8	1.310 (7)
Mo3—O9B	1.952 (3)	C3—N7	1.322 (6)
Mo4—O9B	1.941 (3)	N7—H7A	0.8800
Mo4—O10B	1.959 (3)	N7—H7B	0.8800
Mo5—O10B	1.895 (3)	N8—H8A	0.8800
Mo5—O11B	2.058 (3)	N8—H8B	0.8800
Mo6—O11B	2.075 (4)	N9—H9A	0.8800
Mo6—O12B	1.894 (4)	N9—H9B	0.8800
Mo1—O14T	1.706 (3)	C4—N12	1.320 (12)
Mo1—O13T	1.735 (3)	C4—N10	1.340 (10)
Mo2—O15T	1.702 (3)	C4—N11	1.387 (10)
Mo2—O16T	1.713 (3)	N10—H10A	0.893 (17)
Mo3—O17T	1.705 (4)	N11—H11A	0.898 (18)
Mo3—O18T	1.706 (4)	N12—H12A	0.90 (2)
Mo4—O19T	1.704 (3)	N12—H12B	0.90 (2)
Mo4—O20T	1.706 (3)	O1W—H1AW	0.94 (2)
Mo5—O22T	1.708 (3)	O1W—H1BW	0.95 (2)
Mo5—O21T	1.719 (3)	O2W—H2AW	0.94 (2)
Mo6—O23T	1.710 (3)	O2W—H2BW	0.95 (2)
Mo6—O24T	1.732 (3)	O3W—H3AW	0.93 (2)
O2C—H2	0.96 (2)	O3W—H3BW	0.94 (2)
O3C—H3	0.96 (2)	O4W—H4AW	0.95 (2)
O4C—H4	0.95 (2)	O4W—H4BW	0.95 (2)
			
Mo1—O1C—Mo2	95.79 (12)	O21T—Mo5—O4C	86.44 (14)
Mo2—O2C—Mo3	93.64 (11)	O10B—Mo5—O4C	72.56 (12)
Mo4—O3C—Mo3	93.75 (12)	O11B—Mo5—O4C	85.46 (12)
Mo5—O4C—Mo4	92.64 (11)	O5C—Mo5—O4C	72.30 (12)
Mo6—O5C—Mo5	102.87 (13)	O23T—Mo6—O24T	105.40 (16)
Mo6—O6C—Mo1	91.14 (12)	O23T—Mo6—O12B	101.61 (17)
Mo1—O7B—Mo2	111.71 (15)	O24T—Mo6—O12B	101.99 (16)
Mo3—O8B—Mo2	119.36 (16)	O23T—Mo6—O11B	96.50 (16)
Mo4—O9B—Mo3	119.39 (17)	O24T—Mo6—O11B	89.87 (15)
Mo5—O10B—Mo4	120.02 (16)	O12B—Mo6—O11B	154.72 (14)
Mo5—O11B—Mo6	108.97 (15)	O23T—Mo6—O5C	93.14 (14)
Mo6—O12B—Mo1	116.75 (17)	O24T—Mo6—O5C	155.73 (14)
O1C—Pt1—O5C	99.68 (14)	O12B—Mo6—O5C	89.14 (13)
O1C—Pt1—O6C	84.05 (13)	O11B—Mo6—O5C	72.28 (12)
O5C—Pt1—O6C	83.21 (12)	O23T—Mo6—O6C	166.99 (14)
O1C—Pt1—O4C	177.35 (12)	O24T—Mo6—O6C	87.60 (14)
O5C—Pt1—O4C	82.27 (13)	O12B—Mo6—O6C	75.67 (13)
O6C—Pt1—O4C	97.99 (13)	O11B—Mo6—O6C	82.71 (13)
O1C—Pt1—O2C	82.64 (13)	O5C—Mo6—O6C	74.21 (11)
O5C—Pt1—O2C	177.41 (13)	Pt1—O1C—Mo1	104.21 (13)
O6C—Pt1—O2C	98.20 (13)	Pt1—O1C—Mo2	103.95 (14)
O4C—Pt1—O2C	95.37 (13)	Pt1—O2C—Mo2	101.92 (13)
O1C—Pt1—O3C	95.12 (13)	Pt1—O2C—Mo3	103.51 (13)
O5C—Pt1—O3C	95.66 (12)	Pt1—O2C—H2	116 (4)
O6C—Pt1—O3C	178.46 (12)	Mo2—O2C—H2	113 (4)
O4C—Pt1—O3C	82.87 (13)	Mo3—O2C—H2	125 (4)
O2C—Pt1—O3C	82.96 (13)	Pt1—O3C—Mo4	103.59 (13)
O14T—Mo1—O13T	105.87 (16)	Pt1—O3C—Mo3	102.74 (13)
O14T—Mo1—O12B	99.01 (16)	Pt1—O3C—H3	108 (4)
O13T—Mo1—O12B	98.14 (15)	Mo4—O3C—H3	124 (4)
O14T—Mo1—O7B	100.03 (16)	Mo3—O3C—H3	122 (4)
O13T—Mo1—O7B	95.86 (15)	Pt1—O4C—Mo5	100.53 (13)
O12B—Mo1—O7B	152.25 (14)	Pt1—O4C—Mo4	102.74 (13)
O14T—Mo1—O1C	93.17 (14)	Pt1—O4C—H4	124 (5)
O13T—Mo1—O1C	160.02 (14)	Mo5—O4C—H4	109 (5)
O12B—Mo1—O1C	84.50 (12)	Mo4—O4C—H4	122 (5)
O7B—Mo1—O1C	74.48 (12)	Pt1—O5C—Mo6	103.69 (13)
O14T—Mo1—O6C	165.12 (14)	Pt1—O5C—Mo5	104.87 (14)
O13T—Mo1—O6C	88.14 (14)	Pt1—O6C—Mo6	98.17 (12)
O12B—Mo1—O6C	73.55 (13)	Pt1—O6C—Mo1	98.14 (13)
O7B—Mo1—O6C	83.11 (12)	Pt1—O6C—O6C^i^	120.8 (2)
O1C—Mo1—O6C	73.54 (11)	Mo6—O6C—O6C^i^	121.27 (19)
O15T—Mo2—O16T	107.06 (18)	Mo1—O6C—O6C^i^	120.85 (19)
O15T—Mo2—O8B	98.04 (15)	Pt1—O6C—H6	120.8 (2)
O16T—Mo2—O8B	100.65 (16)	Mo6—O6C—H6	121.27 (19)
O15T—Mo2—O7B	100.70 (15)	Mo1—O6C—H6	120.85 (19)
O16T—Mo2—O7B	96.07 (16)	O6C^i^—O6C—H6	0.00 (18)
O8B—Mo2—O7B	149.93 (14)	Mo5—O11B—H11	118 (7)
O15T—Mo2—O1C	94.74 (15)	Mo6—O11B—H11	125 (7)
O16T—Mo2—O1C	156.95 (15)	N2—C1—N1	123.5 (6)
O8B—Mo2—O1C	83.19 (13)	N2—C1—N3	119.2 (7)
O7B—Mo2—O1C	72.05 (12)	N1—C1—N3	117.0 (6)
O15T—Mo2—O2C	163.07 (14)	C1—N1—H1A	120.0
O16T—Mo2—O2C	88.12 (15)	C1—N1—H1B	120.0
O8B—Mo2—O2C	71.19 (12)	H1A—N1—H1B	120.0
O7B—Mo2—O2C	84.67 (12)	C1—N2—H2A	120.0
O1C—Mo2—O2C	71.47 (12)	C1—N2—H2B	120.0
O17T—Mo3—O18T	106.36 (19)	H2A—N2—H2B	120.0
O17T—Mo3—O8B	98.10 (16)	C1—N3—H3A	120.0
O18T—Mo3—O8B	102.14 (16)	C1—N3—H3B	120.0
O17T—Mo3—O9B	100.99 (16)	H3A—N3—H3B	120.0
O18T—Mo3—O9B	97.16 (16)	N4—C2—N5	119.7 (5)
O8B—Mo3—O9B	147.71 (13)	N4—C2—N6	120.0 (5)
O17T—Mo3—O2C	160.66 (16)	N5—C2—N6	120.3 (5)
O18T—Mo3—O2C	91.76 (16)	C2—N4—H4A	120.0
O8B—Mo3—O2C	70.90 (12)	C2—N4—H4B	120.0
O9B—Mo3—O2C	82.96 (12)	H4A—N4—H4B	120.0
O17T—Mo3—O3C	92.54 (15)	C2—N5—H5A	120.0
O18T—Mo3—O3C	159.28 (16)	C2—N5—H5B	120.0
O8B—Mo3—O3C	83.03 (13)	H5A—N5—H5B	120.0
O9B—Mo3—O3C	70.38 (13)	C2—N6—H6A	120.0
O2C—Mo3—O3C	70.78 (11)	C2—N6—H6B	120.0
O19T—Mo4—O20T	107.04 (16)	H6A—N6—H6B	120.0
O19T—Mo4—O9B	100.65 (15)	N9—C3—N8	121.1 (5)
O20T—Mo4—O9B	97.05 (15)	N9—C3—N7	118.8 (5)
O19T—Mo4—O10B	99.01 (15)	N8—C3—N7	120.1 (5)
O20T—Mo4—O10B	101.47 (15)	C3—N7—H7A	120.0
O9B—Mo4—O10B	147.60 (13)	C3—N7—H7B	120.0
O19T—Mo4—O3C	92.83 (14)	H7A—N7—H7B	120.0
O20T—Mo4—O3C	158.72 (14)	C3—N8—H8A	120.0
O9B—Mo4—O3C	71.24 (13)	C3—N8—H8B	120.0
O10B—Mo4—O3C	82.34 (13)	H8A—N8—H8B	120.0
O19T—Mo4—O4C	161.33 (14)	C3—N9—H9A	120.0
O20T—Mo4—O4C	90.53 (14)	C3—N9—H9B	120.0
O9B—Mo4—O4C	82.92 (12)	H9A—N9—H9B	120.0
O10B—Mo4—O4C	70.65 (12)	N12—C4—N10	120.6 (9)
O3C—Mo4—O4C	70.80 (11)	N12—C4—N11	126.3 (9)
O22T—Mo5—O21T	106.73 (18)	N10—C4—N11	112.7 (7)
O22T—Mo5—O10B	101.18 (15)	C4—N10—H10A	155 (2)
O21T—Mo5—O10B	103.69 (15)	C4—N12—H12A	113 (7)
O22T—Mo5—O11B	96.63 (15)	C4—N12—H12B	122 (8)
O21T—Mo5—O11B	90.77 (15)	H12A—N12—H4AW	106 (10)
O10B—Mo5—O11B	152.59 (14)	H12B—N12—H4AW	20 (10)
O22T—Mo5—O5C	95.73 (15)	H1AW—O1W—H1BW	100 (6)
O21T—Mo5—O5C	152.95 (14)	H2AW—O2W—H2BW	116 (8)
O10B—Mo5—O5C	86.07 (13)	H3AW—O3W—H3BW	92 (8)
O11B—Mo5—O5C	71.47 (12)	H4AW—O4W—H4BW	103 (10)
O22T—Mo5—O4C	166.58 (15)		

Symmetry code: (i) −*x*+3/2, −*y*+1/2, −*z*+1.

### Hydrogen-bond geometry (Å, º)

**Table:**

*D*—H···*A*	*D*—H	H···*A*	*D*···*A*	*D*—H···*A*
O2*C*—H2···O24*T*^i^	0.96 (2)	1.61 (2)	2.578 (5)	179 (6)
O3*C*—H3···O2*W*	0.96 (2)	1.69 (3)	2.622 (6)	164 (7)
O4*C*—H4···O13*T*^i^	0.95 (2)	1.63 (2)	2.568 (5)	173 (9)
O6*C*—H6···O6*C*^i^	1.27	1.27	2.532 (6)	180
O11*B*—H11···O7*B*^i^	0.95 (2)	1.74 (2)	2.679 (5)	173 (10)
N1—H1*B*···O1*C*	0.88	2.05	2.864 (6)	154
N1—H1*A*···O3*W*	0.88	2.33	2.973 (9)	130
N2—H2*A*···O18*T*^ii^	0.88	2.08	2.940 (7)	165
N2—H2*B*···O19*T*^iii^	0.88	2.22	3.043 (6)	155
N3—H3*B*···O8*B*	0.88	2.04	2.874 (7)	157
N3—H3*A*···O2*W*^iii^	0.88	2.25	2.979 (9)	140
N4—H4*B*···O14*T*^iv^	0.88	2.09	2.944 (6)	164
N4—H4*A*···O24*T*^i^	0.88	2.48	3.006 (6)	119
N5—H5*A*···O16*T*	0.88	2.06	2.890 (6)	157
N5—H5*B*···O21*T*^v^	0.88	2.18	2.973 (5)	149
N6—H6*A*···O15*T*^iv^	0.88	2.19	2.894 (6)	136
N6—H6*B*···O21*T*^v^	0.88	2.59	3.281 (6)	136
N7—H7*B*···O19*T*	0.88	2.40	2.936 (5)	119
N7—H7*A*···O1*W*	0.88	2.11	2.927 (6)	154
N8—H8*B*···O13*T*^vi^	0.88	2.39	3.006 (6)	128
N8—H8*A*···O23*T*^vii^	0.88	2.04	2.918 (6)	178
N9—H9*A*···O22*T*^vii^	0.88	2.21	2.938 (7)	140
O1*W*—H1*AW*···O9*B*	0.94 (2)	2.20 (5)	2.916 (5)	132 (5)
O1*W*—H1*BW*···O17*T*^viii^	0.95 (2)	1.85 (3)	2.783 (5)	166 (6)
O2*W*—H2*BW*···O4*W*^ii^	0.95 (2)	2.24 (7)	2.902 (12)	126 (6)
O3*W*—H3*BW*···O9*B*^ii^	0.94 (2)	2.35 (8)	3.029 (7)	128 (8)

Symmetry codes: (i) −*x*+3/2, −*y*+1/2, −*z*+1; (ii) *x*, *y*−1, *z*; (iii) −*x*+1, −*y*, −*z*+1; (iv) *x*, *y*+1, *z*; (v) *x*, −*y*+1, *z*+1/2; (vi) *x*−1/2, −*y*+1/2, *z*−1/2; (vii) −*x*+1, *y*+1, −*z*+1/2; (viii) −*x*+1, −*y*+1, −*z*+1.
